# *Listeria monocytogenes* Survey in Cubed Cooked Ham Packaged in Modified Atmosphere and Bioprotective Effect of Selected Lactic Acid Bacteria

**DOI:** 10.3390/microorganisms8060898

**Published:** 2020-06-15

**Authors:** Lucilla Iacumin, Giorgia Cappellari, Andrea Colautti, Giuseppe Comi

**Affiliations:** Dipartimento di Scienze AgroAlimentari, Ambientali e Animali, Università degli Studi di Udine, Via Sondrio 2/a, 33100 Udine, Italy; lucilla.iacumin@uniud.it (L.I.); giorgia.cappellari@spes.uniud.it (G.C.); andrea.colautti@uniud.it (A.C.)

**Keywords:** cooked cubed ham, bioprotective, *Listeria monocytogenes*, bioprotection

## Abstract

The aim of this work was to study the presence of *Listeria monocytogenes*, as well as the potential activity of two bioprotective cultures (Lyocarni BOX-74 and Lyocarni BOX-57), versus a mix of three *L. monocytogenes* strains that were intentionally inoculated in cooked cubed ham, packaged in Modified Atmosphere Packaging and stored at different temperatures. The bioprotective cultures limit *L. monocytogenes* growth in cubed cooked ham stored either at 4 °C for 60 days and at 4 °C for 20 days and at 8 °C for 40 days. The inhibition at 8 °C is particularly useful for industrial cooked meat products, considering there are often thermal abuse conditions (8 °C) in the supermarkets. Both the starters can eliminate *L. monocytogenes* risk and maintain the products safe, despite the thermal abuse conditions. In addition, both culture starters grew without producing perceptible sensory variations in the samples, as demonstrated by the panel of the untrained tasters. The bioprotective LAB produced neither off-odours and off-flavours, nor white/viscous patinas, slime, discoloration or browning. Therefore, according to the obtained data, and despite the fact that cooked cubed ham did not show pH ≤ 4.4 or a_w_ ≤ 0.92, or pH ≤ 5.0 and a_w_ ≤ 0.94, as cited in the EC Regulation 2073/2005. It can be scientifically stated that cubes of cooked ham with the addition of bioprotective starters cultures do not constitute a favourable substrate for *L. monocytogenes* growth. Consequently, these products can easily fall into category 1.3 (ready-to-eat foods that are not favourable to *L. monocytogenes* growth, other than those for infants and for special medical purposes), in which a maximum concentration of *L. monocytogenes* of 100 CFU g^−1^ is allowed.

## 1. Introduction

*Listeria monocytogenes* can cause fatal disease (30–40%) in foetuses, infants, pregnant women, elderly subjects and immunocompromised individuals with cancer, kidney disease, heart disease or AIDS; subject to organ transplants; and/or treated with immunosuppressants [1,2,3]. The incidence of the disease caused by *L. monocytogenes*, which is named listeriosis, is decreasing and varies every year. In the US, where control is more robust, an annual incidence of 0.7 cases/100,000 inhabitants, with a mortality rate greater than 40%, is estimated [1]. Listeriosis is also widespread in Europe, and although there are slight variations, an incidence of 0.48 cases/100,000 inhabitants is estimated, with a mortality rate of approximately 13.7% [4,5].

*L. monocytogenes* is a microorganism of environmental origin and is isolated from many foods, such as milk and dairy products, fresh and processed meats, fresh and processed (smoked) fish products, vegetables and fruit [4,5,6,7,8,9,10]. By means of biofilm production, *L. monocytogenes* can also contaminate inert surfaces, such as stainless steel, polyethylene and rubber [11,12,13,14,15]. In particular, damp and cold surfaces can provide favourable conditions for the survival, and even the growth of *L. monocytogenes* [14,15]. In fact, *L. monocytogenes* grows without any problem in raw or processed food, because does not have great nutrition or oxygen demands and grows well at 4 °C. In fact, it is well-known that, despite its initial level of contamination, *L. monocytogenes* can quickly reach high concentration levels in some refrigerated ready-to-eat (RTE) products [1,2,16]. The growth of *L. monocytogenes* can be particularly dynamic in foods stored at extreme temperatures, which often occur in both the food trade and domestic refrigerators [17,18]. Among RTE products, cooked ham can be contaminated with *L. monocytogenes* [19,20,21], although cooking and subsequent pasteurisation after final packaging eliminate its presence [19,20,21]. However, *L. monocytogenes* can recontaminate cooked ham during slicing and dicing to obtain cubes used in culinary recipes. To eliminate this recontamination, post-processing technologies are often performed in cleanrooms, where the environment is thoroughly sanitised and the air is filtered, which consequently greatly reduce the spread of *L. monocytogenes* through atmospheric dust. Moreover, slicer equipment is cleaned and disinfected, while workers are trained and wear additional sterile coveralls over their work clothes. Therefore, these measures allow for reduction in contamination. Despite the above-mentioned preventive systems, *L. monocytogenes* is often isolated either in pre-sliced or in cooked cubed ham, and the cause of the recontamination is facilitated by the contact surfaces of the machinery or from the air [19,20,21]. Nonetheless, recontamination cannot occur from the cooked ham bars used for slicing or dicing, because they are cooked in moulds, packaged and pasteurised. All Italian factories use the following protocol: brined pork meat is stuffed into plastic bags, placed in mould-like bars and cooked at up to 70–73 °C (the cooking process lasts for 7 h; 1 h per kg product); then, after cooling at 4 °C, the product is stored for 4–5 days at 4 ± 2 °C, pasteurised at 85 °C for 15 min, cooled, subjected to dicing, and packed in Modified Atmosphere Packaging (MAP) (100 g product each). Therefore, contamination can only take place in dicing machines or slicers. Moreover, this equipment has often been associated with *L. monocytogenes* contamination of retail RTE meat products [20,21,22,23,24,25,26,27]. *L. monocytogenes* was isolated by swabs from 13% of slicer blades used in different butchers [28], and from 10% of delicatessen knives and supermarket slicers [10,22,29,30,31,32]. Contamination occurs from the slicer to the product, and vice versa [22,31,33], thereby causing cross-contamination. In this regard, thorough cleaning and disinfection protocols can prevent the formation of *L. monocytogenes* biofilms, which usually grow over time, through continuous and prolonged cross-contamination. It is known that the presence of this pathogen on both products and contact surfaces [34], the composition of the food [31,35,36,37], the cutting force and the speed of the blade [38], and other factors, such as temperature [22], surface topography [31] and contact time [30,35,39], can contribute to the recontamination of cooked charcuterie products. However, the level of this accidental contamination is usually low, at less than 10 CFU g^−1^ or cm^−2^ [18,22,31,33]. Despite this low initial concentration, sliced or diced cooked ham can represent a risk for consumers, because *L. monocytogenes* can reach high concentrations during their shelf lives at 4 °C of 30 and 60 days, respectively. In particular, in the case of thermal abuse (6–8 °C) during storage at the supermarket, *L. monocytogenes* may grow and reach dangerous concentrations for the consumer, considering that slices of cooked ham are eaten as is, and that cubes are not always cooked before consumption. Recently, to avoid the presence or growth of *L. monocytogenes* in meat products, in addition to the application of good production hygiene standards, bioprotective cultures or natural preservatives, such as plant or animal (chitosan and propolis) and microbial extracts or metabolites (lysozyme, nisin and other bacteriocins), have been used [40,41,42,43,44]. Since the use of plant and animal extracts can often change the smell, taste and texture of food [45], there has been an increase in the direct use of bacteriocins with antimicrobial effects or bioprotective cultures, because of their antagonistic effect and production of organic acids or bacteriocins against *L. monocytogenes* [46,47]. These bacteriocin inhibition effects are likely caused by different actions, such as competition for nutrients, as well as organic acid and bacteriocin production, in addition to the “hurdle” parameters. Strains of *Lactobacillus sakei, L. casei, L. brevis, L. curvatus, L. plantarum* and *Carnobacterium* spp. isolated from meat products frequently produce bacteriocins or bacteriocin-like compounds [48,49,50,51,52,53]. In particular, these strains have good antilisterial effects, and are therefore used as bioprotective cultures in European meat products [48,49,50,51,52,53].

Currently, evidence on the use of bioprotective cultures against the presence and growth of *L. monocytogenes* in cooked cubed ham remains scarce. Therefore, the aim of this work was to evaluate the use of two selected commercial bioprotective starter cultures, to eliminate or prevent the development of *L. monocytogenes* intentionally added to cooked cubed ham stored for 60 days (end shelf life) at 4 °C, and under thermal abuse conditions (8 °C).

## 2. Materials and Methods

### 2.1. L. monocytogenes Monitoring in Cooked Cubed Ham Packages

One hundred and eighty (180) samples of cooked cubed ham, placed in Modified Atmosphere Packaging (MAP) of 100 g of product produced by 3 Italian factories (60 each), were collected from local supermarkets and analysed in an epidemiological study to evaluate the frequency of the presence of *L. monocytogenes*.

Samples belonged to 6 different lots of production. Each lot included 10 samples, 5 of which were analysed immediately after purchase, whereas the remaining 5 were stored at 4 ± 2 °C and analysed at the end of their shelf life (60 days). *L. monocytogenes* was determined using the ISO 11290-1 method [54]. The positive samples were also evaluated by the ISO 11290-2 method [54].

### 2.2. Cooked Cubed Ham Package Preparation for a Bioprotective Study

Cooked ham was produced by one of the main Italian factories. The production steps included the use of various trimmings of the pork leg (75%) and saline (water, natural spices and their extracts, dextrose, sodium ascorbate, carrageenan, sodium chloride and sodium nitrite-E250, the concentrations of which remained confidential). The brined pork meat was stuffed into plastic bags, which represented the final package, and was then cooked up to 73 °C (the cooking process lasted for 7 h; 1 h per kg product). Then, after cooling at 4 °C, the product was stored for 4 days at 4 ± 2 °C, pasteurised at 85 °C for 15 min and, after cooling, unpacked and subjected to dicing, to prepare the samples for the trials.

### 2.3. Preparation of the Inoculum of Listeria monocytogenes and Bioprotective Starter Cultures

The inoculum consisted of 3 strains of different *Listeria monocytogenes* strains: *L. monocytogenes* NCTC 10887 (serotype 1/2b), *L. monocytogenes* 9Di4A (serotype 4b) isolated from the meat matrix and *L. monocytogenes* 11Di4A, which was isolated from human samples, and is responsible for invasive listeriosis. Four millilitres of an overnight culture in Brain Heart Infusion broth (Oxoid, Italy) of each strain as subjected to centrifugation at 13.400 rpm for 10 min at room temperature, after which, the pellets were resuspended in saline-peptone water (Peptone, 1 g, Oxoid, Italy; NaCl, 30 g, Sigma-Aldrich, Italy; distilled H_2_O, 1000 mL; Aw 0.97). Then, the suspensions were standardised at an optical density at 600 nm [OD_600_] of 0.1, and their microbial counts were evaluated. In particular, serial dilutions were carried out in sterile saline-peptone water, and 0.1 mL of each dilution was spread in Petri dishes containing Palcam Agar Base (Oxoid, Italy). Incubation was performed at 37 °C for 48 h, and the colonies were counted. Each suspension contained approximately 7 log CFU mL^−1^. At this step, 1 mL of each standardised suspension was combined, and inoculation was performed by spraying a final concentration of 2 log CFU g^−1^ on the cooked cubed ham packages.

Two different bioprotective cultures consisting of freeze-dried lactic acid bacteria were used: Lyocarni BOX-74 and Lyocarni BOX-57 (Sacco srl, Via Alessandro Manzoni 29/A, 22071 Cadorago, CO, Italy). Lyocarni BOX-74 contained *Carnobacterium divergens*, *Carnobacterium maltaromaticum* and *Lactobacillus sakei*, whereas Lyocarni BOX-57 contained the *Carnobacterium divergens*, *Carnobacterium maltaromaticum* and *Lactobacillus sakei* bacteriocin producers). Both of the cultures were chosen because of their fast growth at 4 °C, and because they are selected and commercially sold, due to their ability to prevent spoilage and pathogenic microorganisms in meat products. Before usage, the effective concentration was checked. Ten (10) grams of freeze-dried cultures were diluted 1:10 in sterile peptone water (NaCl 2%; Aw 0.98) and homogenised. After setting up the decimal dilution in peptone water, counts were performed in deMan Rogosa Sharpe medium (MRS, Oxoid, Italy) using a double layer method. Plates were incubated at 37 °C for 48–72 h, and the colonies were counted. The concentration of both bioprotective starters cultures was 11 log CFU g^−1^. After dilution, each starter was inoculated by spraying the cooked cubed ham with a final concentration of 5 log CFU g^−1^.

### 2.4. Inoculation Design

In parallel, a total of 6 trials were set up as described below:

(A) Control trial (not inoculated, NI);
(B) Samples inoculated with the Sacco BOX-74 bioprotective starter culture and *L. monocytogenes* (BOX74 + LM);
(C) Samples inoculated with only the Sacco BOX-74 bioprotective starter culture (BOX74);
(D) Samples inoculated with only *L. monocytogenes* (LM);
(E) Samples inoculated with the Sacco BOX-57 bioprotective starter culture and *L. monocytogenes* (BOX57 + LM);
(F) Samples inoculated with only the Sacco BOX-57 bioprotective starter culture (BOX57).

For each trial, 33 samples, each of which consisted of 100 g of cooked cubed ham (prepared as described in Section 2.1), were prepared and inoculated following the scheme described above.

Then, the cooked cubed ham (100 g each) was packaged using trays with top (PET/PE/EVOH/PE) and bottom films (PVC/EVOH/PE) in Modified Atmosphere Packaging (MAP), consisting of N_2_ (55%) and CO_2_ (45%). The inoculated samples were left for 2 h at room temperature to favour adhesion of the microorganism to the cubes. Then, two different storage temperatures were tested: 4 °C for the entire shelf life (60 days), 4 °C for the first 20 days, and 8 °C for the remaining shelf life (thermal abuse, 40 days). Analyses were performed on three biological replicates at 0, 10, 20, 30, 40, 50 and 60 days. In addition, duplicate technical replicates were performed for each of the 3 biological replicates per trial stored at both temperatures.

### 2.5. Microbiological Analysis

Each sample (100 g) was completely diluted with saline-peptone water (peptone, 1 g; NaCl, 7 g; distilled H_2_O, 1000 mL) at a 1:1 ratio in Stomacher bags. After homogenisation for 2 min in Stomacher bags (PBI, Italy), decimal dilutions were prepared. Lactic acid bacteria (LAB) were counted by inoculation of 1 mL of each serial dilution in MRS medium (Oxoid, Italy) using the double layer technique. The plates were incubated at 37 °C for 2 days, after which the grown colonies included in the agar medium were counted. The total bacterial count (CBT) was monitored by plating 0.1 mL of each serial dilution on Plate Count Agar (Oxoid, Italy), followed by incubation at 25 °C for 2 days. *Listeria* spp. and *L. monocytogenes* were determined using the ISO 11290-2 method [54].

### 2.6. Physicochemical Analysis

Samples from trials NI, BOX74 and BOX57 were also subjected to physicochemical analysis as follows: (a) determination of water activity (a_w_), carried out at each sampling point by the use of an AquaLab device (Decagon, USA), according to the manufacturer’s instructions, and after appropriate calibration; (b) pH determination using a glass electrode mounted on a pH meter (Crison Basic 20, Italy); and (c) colour determination using the Minolta Chromameter CR-200 and CIE Lab system after calibration. Ten different positions on the surface of each sample were immediately evaluated after opening the tray. In particular, parameters a*, b*, L*, and ΔE were evaluated [55]. Moisture, proteins, fat, sugar and ash were determined by AOAC [56].

### 2.7. Sensory Analysis

Ten additional samples of the (a) control trial (not inoculated, NI); (b) samples inoculated with only the Sacco BOX-74 bioprotective starter culture (BOX74); and (c) samples inoculated with only the Sacco BOX-57 bioprotective starter culture (BOX57) were prepared for sensory analysis. The analysis was performed by 12 untrained panellists, representing real consumers. The samples were evaluated by tasters, who were asked to identify the products in ascending order from worst to best, keeping in mind the following parameters established by Baublis et al. [57] and Vàlcovà et al. [58]: smell (fermented, rancid), flavour (sweet, salty, fresh pungent, meat and rancid) and aroma (ammoniacal, sweet, salty and bitter). Panel members were trained according to the consensus method, in six to eight sessions of at least 90 min each. During each session, subjects were trained, using a roundtable discussion, to achieve (a) lexicon development, (b) training in intensity scaling and (c) reference association with aroma, flavour and mouthfeel attributes. The scaling of attributes on unstructured line scales was practiced, to calibrate the panel until a consensus was reached amongst the panellists.

### 2.8. Statistical Analysis

Data were subjected to two analyses. In the first, the differences between means within storage day were tested by one-way ANOVA, where the experimental group was considered as fixed factor. In the second analysis, the differences between means within experimental group were tested by one-way ANOVA, where storage day was considered as fixed factor. For both analyses, Tukey’s test was used as post-hoc test (*p* < 0.05).

## 3. Results

### 3.1. Epidemiology of L. monocytogenes in Cooked Cubed Ham Packages

The physicochemical characteristics demonstrated the high nutritional value of cooked cubed ham. The results represent the means of all the samples. As shown in Table 1, the samples were rich in proteins, and included low concentrations of fat and sugars. No significant differences were observed among the lots of production and brands, thereby demonstrating the use of the same processes and production technology. These characteristics make the product excellent for human health and diet. The study demonstrated the presence of *Listeria monocytogenes* in cooked cubed ham (Table 2). *Listeria monocytogenes* was isolated in eight out of the 180 investigated samples (4.44%), as well as in some samples analysed at either 0 or 60 days. At time 0, the pathogen was only detected in two samples after using the enrichment method. At 60 days, *L. monocytogenes* was detected in five samples by enrichment, and in one sample by direct dilution at a concentration of 2.1 log CFU/g. The observed values exceeded the limit proposed by Reg. CE 2073/05 [59]. The results demonstrated that *L. monocytogenes* is widespread and contaminates food randomly. The distribution of *Listeria monocytogenes* is not homogeneous, and depends on the contaminated environment, considering that the cooked ham bars were pasteurised at 85 °C for 5 min, cooled, unpackaged and then diced. Finally, contamination was found in each lot from the factories.

### 3.2. Physicochemical Characteristics of Cooked Cubed Ham

The cubed cooked hams were produced in one of the main Italian factories, but the technology is largely used in Italy. The a_w_ value remained within the range of 0.986 ± 0.004 and 0.994 ± 0.003 for the entire monitored period in all trials (Table 3). No significant differences were observed in the samples stored at 4 °C (*p* > 0.05). Conversely, a significant difference was observed among the a_w_ values of the samples stored for 20 days at 4 °C, and then at 8 °C for the remaining 40 days (*p* < 0.05). In particular, the significance is most evident at 0, 30 and 50 days. Comparing the a_w_ at level of both temperatures and days storage significative differences were observed at day 30, 40 and 50. In fact, this difference cannot be considered a variation due to the storage temperature, but instead, is an intrinsic variation of the analysed samples, which were different at each analysed time. Moreover, at 50 and 60 days, the a_w_ values were similar to those assessed at 0, 10, 20 and 30 days.

The trends of the pH of the inoculated and non-inoculated samples of cooked cubed ham are shown in Table 4. At 30 days after production, a decrease in pH can be seen in both the inoculated and uninoculated samples. This decrease is related to the metabolism of LAB, which are considered to be mainly responsible for acidification. The presence of the bioprotective starters produced a significant decrease in pH values, particularly in products stored first at 4 °C, and then at 8 °C. In fact, the observed variation was particularly significant (*p* < 0.05) in samples inoculated with the bioprotective cultures, as well as both in samples stored at 4 °C for the entire period, and those stored for 20 days at 4 °C, and then 40 days at 8 °C. In the uninoculated samples, a significant pH variation was observed at 30 and 40 days in samples stored at 4 °C, as well as at 30 days in samples stored at 4 °C and then at 8 °C. However, it is possible that the pH variation depends not only on the growth of the bioprotective starter LAB, but also on the samples analysed, which were different at all times. Comparing the pH fate at level of each temperature and each treatment (Table 4), significant differences were observed after 30 days storage (*p* < 0.05). In particular the difference was higher at level of 20 days at 4 °C, and then 40 days at 8 °C storage. It seems that BOX-57 starter produced a higher pH decrease than BOX-74 starter (*p* < 0.05) in samples stored over 30 days at 4 °C, and then 40 days at 8 °C (*p* < 0.05). However, it is possible that the pH variation depended not only on the growth of the bioprotective starter LAB, but also on the samples analysed, which were different at all times.

**Figure 1:**
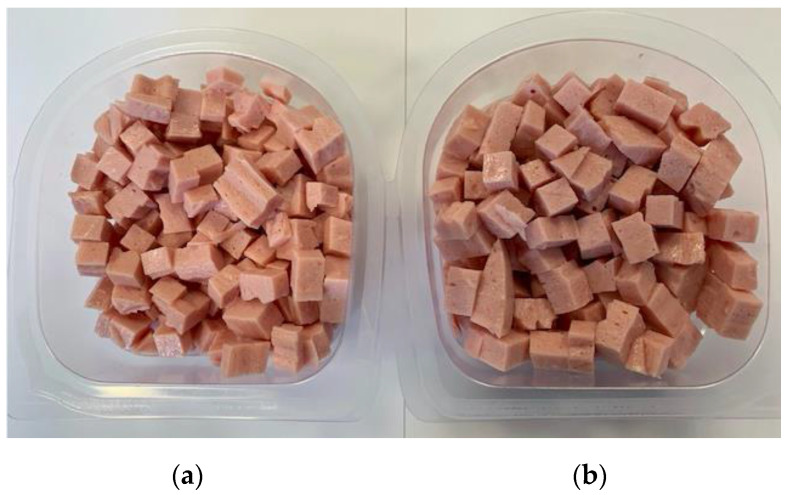
Cooked ham cubes with (**a**) and without (**b**) starter.

Considering the colour changes, there was no significant difference over time among all the investigated samples (Table 5). In fact, the a*, b* and L* values showed wide standard deviations, which influenced the significance (*p* > 0.05), regardless of the storage temperatures and the analysed times. The lack of an important change was due to the MAP system used, which included a low oxygen concentration (less than 0.2%), and, consequently, prevented any oxidation or browning (Figure 1). In addition, starter cultures, which are microaerophilic, produced a reducing potential that limited any oxidation. In any case, the colour variation was not visible to the naked eye (Figure 1), given that the ΔE value was lower than 2.0 [55].

### 3.3. Microbial Evolution and Interaction between Bioprotective Culture and L. monocytogenes

**Figure 2:**
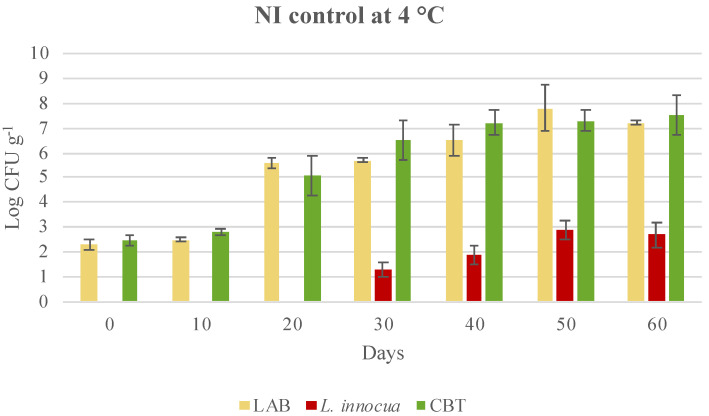
Evolution of microbial population in not-inoculated cubed cooked ham (control—NI), and stored at a controlled and constant temperature of 4 °C till the end of shelf-life. Data were expressed as mean ± standard deviation of the technical (2) and biological replicates (3); LAB, lactic acid bacteria; CBT, total bacterial count.

**Figure 3:**
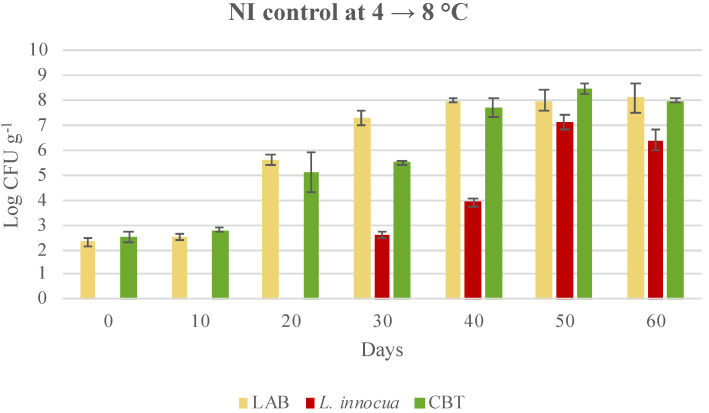
Evolution of microbial population in not-inoculated cubed cooked ham (control—NI) and stored at 4 °C for 20 days and then at 8 °C till the end of shelf-life. Data were expressed as mean ± standard deviation of the technical (2) and biological replicates (3); LAB, lactic acid bacteria; CBT, total bacterial count.

Autochthonous CBT and LAB grew over time and reached final values higher than 7 log CFU g^−1^, independent of the storage temperature (Figure 2 and Figure 3). The growth of both CBT and LAB increased in the samples stored under thermal abuse (4 °C and 8 °C) and exceeded 8 log UFC g^−1^ (Figure 3). These control cooked cubed hams were also naturally contaminated by *L. innocua,* which was not identified at 0, 10 and 20 days of storage, due to being present lower than the detection limit of 10 CFU g^−1^. Subsequently, during storage, *L. innocua* grew to a final concentration of 2.5 ± 0.5 log CFU g^−1^ in NI samples at 4 °C and even reached 7.1 ± 0.3 log CFU g^−1^ in NI samples stored at 4 and then 8 °C, despite the competition of autochthonous LAB (Figure 2 and Figure 3). These data suggest that the shelf life of the cooked cubed ham should not exceed 30–40 days. During a longer shelf life, in the case of accidental contamination, *L. monocytogenes* could grow and reach concentrations dangerous for consumer health.

**Figure 4:**
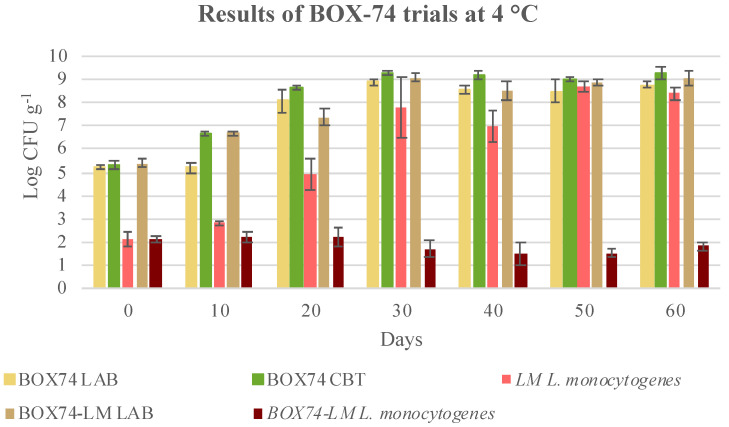
Evolution of microbial population in cubed cooked ham inoculated with BOX-74 starter (BOX74), *Listeria monocytogenes* (LM), and both BOX-74 starter and *L. monocytogenes* (BOX74 + LM) and stored at 4 °C till the end of shelf-life. Data were expressed as mean ± standard deviation of the technical (2) and biological replicates (3); LAB, lactic acid bacteria; CBT, total bacterial count.

Considering the results of the coinoculation of the BOX-74 bioprotective culture and *Listeria monocytogenes* (Figure 4), a significant growth of the bioprotective culture, and a clear inhibition of the inoculated *L. monocytogenes* can be observed.

Given the wide standard deviation observed, *L. monocytogenes* reduction was not statistically significant, (*p* > 0.05) within the same temperature, in respect to that at day 0, although a slightly decreasing trend can be observed over time (Table 6). Vice versa, when the bioprotective culture was not inoculated (LM trial), *L. monocytogenes* grew and reached final values higher than 8 log CFU g^−1^, thereby becoming a serious health risk for consumers, even though these products are usually cooked before consumption.

Therefore, the bioprotective culture was effective. *L. monocytogenes* strains were not completely eliminated, but their growth capability was inhibited. When the bioprotective culture BOX-74 was inoculated in the absence of *L. monocytogenes*, abundant growth was observed and counts close to 9.0 log CFU g^−1^ were observed (Figure 4). Almost the same counts were observed in the case of the co-inoculated samples (BOX74-LM), confirming that an inhibitory effect was shown, or rather, a small increment in the counts of the co-inoculated samples could be observed. The growth of the bioprotective culture, either alone or with *L. monocytogenes* inoculation, was also confirmed by the pH trend, which changed from 6.35 units (day 0) to 5.69 units at the end of shelf life (60 days). The CBT increased over time, and concentrations reached were slightly higher than those of the bioprotective culture. As a matter of fact, the CBT counts included the LAB counts in both the inoculated and non-inoculated samples (natural LAB contamination), which explained the particularly high value obtained. For confirmation, five colonies were isolated for each plate count of PCA, and the presumptive identification of LAB species was performed.

**Figure 5:**
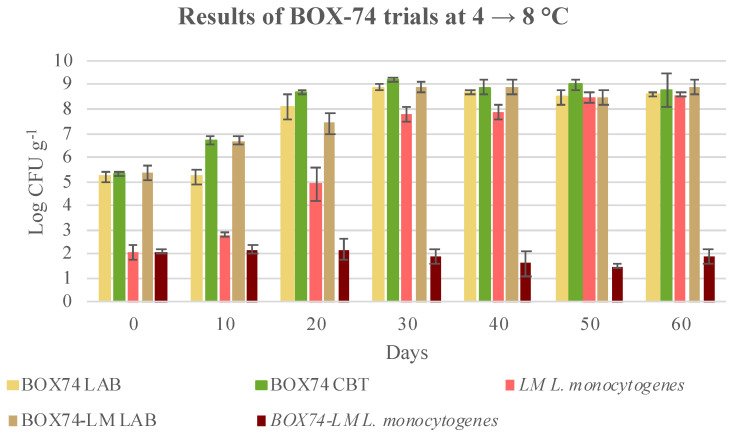
Evolution of microbial population in cubed cooked ham inoculated with BOX-74 starter (BOX74), *Listeria monocytogenes* (LM), both BOX-74 starter and *L. monocytogenes* (BOX74 + LM) and stored at 4 °C for 20 days and then at 8 °C till the end of shelf-life. Data were expressed as mean ± standard deviation of the technical (2) and biological replicates (3); LAB, lactic acid bacteria; CBT, total bacterial count.

In the case of storage under thermal abuse conditions (from 4 to 8 °C), a similar trend was observed (Figure 5). A clear inhibition of the growth of *L. monocytogenes* was obtained, due to the presence of the BOX-74 bioprotective culture. The value of decrease was not significative and was similar to the one observed in the samples stored at 4 °C (*p* > 0.05, Table 6). BOX-74 inhibited *L. monocytogenes* growth, despite the higher temperature maintained for the last 40 days of storage. In the trials in which BOX-74 was not inoculated, *L. monocytogenes* reached final concentrations higher than 8.4 log CFU g^−1^. Therefore, the selected bioprotective culture BOX-74 was effective in preventing the development of 6 logs of *L. monocytogenes*, and resulting in a consistent bacteriostatic effect. Consequently the *L. monocytogenes* concentration remained < 2 log CFU g^−1^, as accepted by Reg. CE 2073/05 [59]. The LAB bioprotective culture, when inoculated in isolation, grew abundantly, and reached values of approximately 8.6 ± 0.1 log CFU g^−1^. These values are not significantly different from those observed in the samples with the BOX-74/*L. monocytogenes* co-inoculation. This finding confirms that the presence of *L. monocytogenes* did not affect the viability of the bioprotective culture. The active metabolism of the bioprotective culture was also demonstrated by the pH trend, which decreased to 5.4 units at 60 days of storage. This value is lower than that observed in samples stored at 4 °C, until the end of shelf life. Thermal abuse (8 °C) increased bioprotective culture activity. CBT increased over time, and reached concentrations similar to those observed for the bioprotective culture.

**Figure 6:**
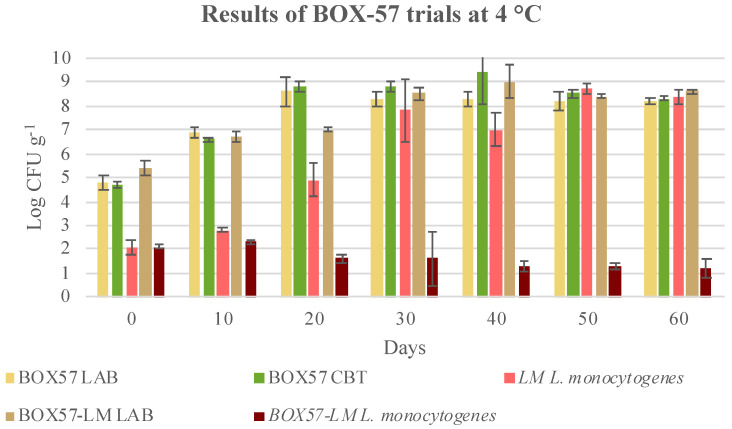
Evolution of microbial population in cubed cooked ham inoculated with BOX-57 starter (BOX57), *Listeria monocytogenes* (LM), and both BOX-57 starter and *L. monocytogenes* (BOX57 + LM) and stored at 4 °C till the end of shelf-life. Data were expressed as mean ± standard deviation of the technical (2) and biological replicates (3); LAB, lactic acid bacteria; CBT, total bacterial count.

**Figure 7:**
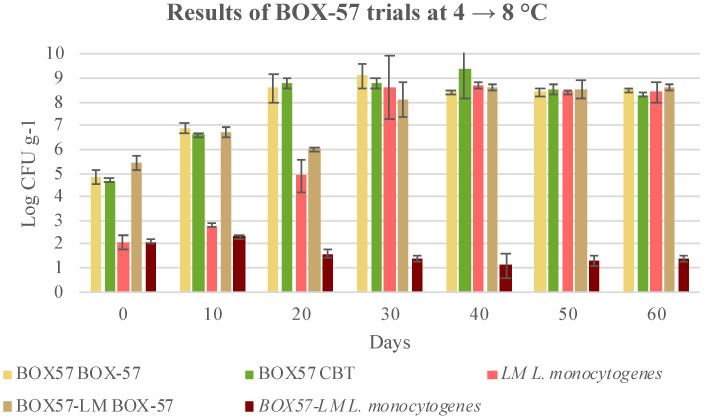
Evolution of microbial population in cubed cooked ham inoculated with BOX-57 starter (BOX57), *Listeria monocytogenes* (LM), and both BOX-57 starter and *L. monocytogenes* (BOX57 + LM) and stored at 4 °C for 20 days and then at 8 °C till the end of shelf-life. Data were expressed as mean ± standard deviation of the technical (2) and biological replicates (3); LAB, lactic acid bacteria; CBT, total bacterial count.

As far as the BOX-57 bioprotective culture is concerned, *L. monocytogenes* growth was inhibited and the level of inhibition was statistically significant (*p* < 0.05), beginning as early as 20 days after the co-inoculation. This inhibitory effect was visible in both trials at 4 °C, and also when the cooked cubed hams packages were stored under thermal abuse conditions (4 to 8 °C) (Figure 6 and Figure 7). This result confirms the higher effectiveness of BOX-57 than BOX-74. On the other hand, *L. monocytogenes* was able to grow and reached final concentrations higher than 8.7 log CFU g^−1^ at 50 days after inoculation in cooked cubed ham, without the addition of the bioprotective culture. Therefore, BOX-57 was also effective and prevented the growth of inoculated *L. monocytogenes* strains. When inoculated alone, BOX-57 grew and reached concentrations slightly higher than 8.0 Log CFU g^−1^ at 4 °C and even greater than 9.0 Log CFU g^−1^ at 4 to 8 °C at 30 days. These values were lower than those reached by the starter inoculated with *L. monocytogenes*. In fact, at 4 °C, the sample grew quickly, even within 40 days, reaching 9.0 Log CFU g^−1^. However, at 50 and 60 days, the concentrations slightly decreased. The development of the starter, either added as a single culture or in a mix with *L. monocytogenes*, was confirmed by the pH trend. CBT increased over time and reached concentrations similar to those of the LAB (Figure 6 and Figure 7).

The storage temperature of approximately 8 °C, which occurred from the 20th day until the end of shelf life, did not favour the development of *L. monocytogenes* in the presence of BOX-57. However, *L. monocytogenes* was not completely eliminated, although its growth was prevented.

Comparing the levels of *L. monocytogenes* reduction in samples treated with BOX-74 and BOX-57, it appears that BOX-57 is more effective. In particular, at 20, 30 and 60 days storage, BOX-57 produced a significative *L. monocytogenes* reduction respect to BOX-74 (*p* < 0.05) in samples stored 20 days at 4 °C and 40 days at 8 °C (Table 6). Conversely, at 4 °C, the significative difference using BOX-57 was observed only at 60 days storage (Table 6). Consequently, it seems that the best performances in *L. monocytogenes* reduction can be obtained by BOX-57 starter.

### 3.4. Sensorial Analysis of Cubed Cooked Ham Samples Treated or not with BOX-74 and BOX-57 Starter Cultures

Sensory analysis was carried out by a panel of non-professional panellists on the uninoculated control samples, as well as the samples inoculated with only BOX-74 and BOX-57. The starters did not profoundly change the sensorial characteristics of the product (Table 7; Figure 1). In fact, cooked cubed ham treated with bioprotective LAB presented neither typical odours and flavours of spoilage nor white/viscous patinas, slime, discoloration or browning. The lack of change was expected, because both starters were selected for their antagonist activity versus spoilage and pathogenic bacteria, without off-odour and off-flavour production. Thirty-one out of 33 control samples did not present any spoilage, with only two (6%) among the samples stored at 8 °C being blown, due to heterofermentative LAB growth. Conversely, none of the samples inoculated with the bioprotective cultures were swollen. The LAB cultures were homofermentative and prevented the development of heterofermentative contaminants by substrate competition. Therefore, starter addition can have a dual purpose, preventing the growth of either *L. monocytogenes* or spoilage bacteria, as represented by the heterofermentative LAB.

The panel did not identify any difference in the colour of the samples, regardless of the presence or absence of the starters or the storage temperatures. The presence of slime, discoloration or browning produced by the spoiler bacteria or by the indigenous LAB has not yet been highlighted (Table 7). The only difference between inoculated or non-inoculated samples was a change in pH; however, this change was not deemed significant by the panellists. The panel identified a slightly acidic, non-disturbing taste in the samples added with the starters, and among these, cooked cubed ham inoculated with the BOX-57 was the most appreciated.

## 4. Discussion

Cooked ham is a meat product that, at the end of production, is usually *L. monocytogenes*-free. The production technology allows for the cooking of brined meat in moulds and its pasteurisation or sterilisation after packaging in aluminium or plastic bags. These treatments eliminate the asporogenous pathogenic microorganisms that may be present in the meat or derived from the production environment after the pre-moulding of ham before final packaging [20,21]. Nevertheless, it is also a good ecosystem for microorganisms that can contaminate the product to make it unsafe. For this reason, the choice of a specific method of preservation that guarantees increasing attention for controlling the shelf life and safety of this new generation of minimally processed ready-to-eat food products of extended durability under refrigerated conditions is fundamental. Cooked cubed ham is produced from pork meat, which is brined, packaged under vacuum, cooked in an oven, pasteurised, and then diced and packaged in MAP. The heat treatments eliminate or reduce asporogenous microorganisms. However, during dicing, recontamination occurs and defines the spoilage flora, which mainly consist of LAB and are responsible for the decay of the shelf life of the product. In addition, pathogenic microorganisms, including *L. monocytogenes*, can re-contaminate the product. *L. monocytogenes* is a ubiquitous microorganism and can be found in various substrates, including meat and meat products. The level of contamination of *L. monocytogenes* is always limited and usually less than 1 CFU g^−1^ [20,21,60]. However, it is possible that during storage in the refrigerators of supermarkets, *L. monocytogenes* may develop and reach values capable of producing illness in the consumer. The growth of *L. monocytogenes* is favoured by the thermal abuse or long shelf lives to which these products are subjected (from 23 to 30 days for cold cuts and to 60 days for cooked cubed ham). Our study demonstrated that dicing permits *L. monocytogenes* to contaminate cooked cubed ham and that its presence in 25 g of product was found in eight out of 180 samples harvested from retail locations. In these specific samples, the concentration of *L. monocytogenes* was found to consistently be < 100 CFU g^−1^. Only in one out of the 180 samples, the concentration of *L. monocytogenes* was detected at a level of 2.1 Log CFU g^−1^ at 60 days of storage. This value demonstrates that *L. monocytogenes* can grow during a long storage period at 4 °C. In Europe, the presence of *L. monocytogenes* has been described in cooked meats [6,7,20,21,26,61]. *L. monocytogenes* was present in 1.65% of cooked ham and in 6.65% of cooked ham slices [26]. Data confirmed that slicing, even if performed in clean rooms with a high level of hygiene, produces contamination or recontamination and, consequently, poses a health risk. Cooked ham is a ready-to-eat (RTE) product, and is normally eaten without cooking; therefore, it can become dangerous to eat if it harbours *L. monocytogenes* [62]. Several studies have been performed to evaluate the survival or growth of this microorganism in RTE meat products [21,63,64,65,66]. Again, mathematical models were also used to describe the behaviour of *L. monocytogenes* in RTE meats, taking into account their intrinsic and extrinsic parameters, such as the pH; acidity; a_w_; salt ratio; presence of nitrite, polyphosphates, lactic acid and diacetate; packaging under vacuum or in a modified atmosphere; and storage temperature [67,68]. Data showed that *L. monocytogenes* can widely grow in cooked ham due to the pH value (>6.0), a_w_ (>0.98), additives (nitrite, sugar, milk powder, proteins), brine and storage times and temperatures used.

Ingredients and additives cannot hinder the microorganism’s growth, and post-processing heat treatments after dicing or slicing cannot be applied. Consequently, to solve the *L. monocytogenes* problem, the use of others innovative technologies based on natural additives (e.g., essential oils) or bioprotective cultures are required [69].

Bioprotective selected starters represent a valid way to eliminate or prevent the development of *L. monocytogenes* in products that support its growth. The use of bioprotective cultures, represented by two types of cultures, including Lyocarni Sacco BOX-74 (*Carnobacterium divergens, Carnobacterium maltaromaticum* and *Lactobacillus sakei*) and Lyocarni BOX-57 (*Carnobacterium divergens; Carnobacterium maltaromaticum* and *Lactobacillus sakei* bacteriocin producer), demonstrated efficiency in inhibiting *L. monocytogenes* growth either at 4 °C, or at 4 °C for 20 days, and then at 8 °C for 40 days. The activity of these bioprotective cultures is mainly based on competition at the substrate level and also, in the case of BOX-57, bacteriocin production.

Microbial starters are usually added to raw meat to promote its ripening or shelf life, because they develop and predominate against pathogenic and spoiler microorganisms. In this work, the added cultures not only inhibited *L. monocyogenes*, but also prevented heterofermentative LAB growth, which was responsible for spoiling some cooked cubed ham by slimes and blown package production. The presence of autochthonous (natural) LAB, as demonstrated in the experiment, is not sufficient to inhibit *L. monocytogenes*. In the control samples, in which only *L. monocytogenes* was inoculated, a quick development of the pathogen was observed. Several authors demonstrated the effectiveness of selected cultures to avoid *L. monocytogenes* growth in cooked meats. Bredholt et al. [52,53] and Schobitz et al. [70] showed that the growth of *L. monocytogenes* can be inhibited by *Lactobacillus sakei* in vacuum packages of sliced cooked ham and by *Carnobacterium piscicola* in vacuum packed meats, respectively. Both strains were isolated by meat product. Vermeiren et al. [71] confirmed the importance of culture selection, and their research demonstrated that only 92% of the isolated LAB strains were able to inhibit *L. monocytogenes* growth. Amezquita and Brashears [61] demonstrated that selected *Pediococcus acidilactici, Lactobacillus casei* and *Lactobacillus paracasei* strains, isolated from meat based RTE products, showed bacteriostatic activity in cooked meats and bactericidal activity towards *L. monocytogenes* in frankfurters stored at 5 °C.

In this case, 2 Log CFU g^−1^ was chosen as the ideal *L. monocytogenes* concentration, and the inoculated samples were then packaged in MAP in plastic trays. This concentration was intentionally high to simulate a high level of recontamination during dicing and packaging. The bioprotective cultures, represented by the *Carnobacterium divergens, C. maltaromaticum* and *Lactobacillus sake* strains, were selected from meats and therefore used in these products. All the strains were able to develop at storage temperatures (4 °C) and compete both in vitro and in situ against *L. monocytogenes*. In particular, BOX-57 contains a *Lactobacillus sakei* strain known to be a bacteriocin producer.

It is well-known that LAB can produce bacteriocins, which are peptides or glycopeptides that contain structures that are closely related to the strain producer. Biochemical and molecular characteristics identify their type [69]. Several bacteriocins isolated from various food sources have been identified, studied and used in meat and meat products, including pediocins, lactocin 705, sonorensin, plantarocins and enterocines [44,69]. Bacteriocins are often purified from LAB and directly inoculated in raw or processed meats. However, direct inoculation of the bacteriocin producer LAB strain is preferred [69]. In recent years, combined techniques have been increasingly used. Bacteriocins are often associated with additives or other technologies (sodium nitrite, heat treatments, high pressures, etc.), which produce a synergistic effect [44,69]. Such combinations can solve many problems related to the presence of *L. monocytogenes* in cooked ham. Wu et al. [44] inhibited *L. monocytogenes* in cooked ham using physicochemical techniques combined with plantaricin.

## 5. Conclusions

The physicochemical parameters of the tested product (a_w_ > 0.98 and pH > 5.0) were optimal for the growth of *L. monocytogenes*. To prevent the growth of *L. monocytogenes*, the use of bioprotective starters is recommended. In fact, the LAB bioprotective cultures grew throughout the storage period, independent of the storage temperature, leading to a significant decrease in the concentration of the pathogen. In all the samples with added bioprotective starter cultures, an increase of *L. monocytogenes* was observed, surpassing a 4 Log development that was conversely observed in the non-inoculated control samples. Moreover, the bioprotective cultures resulted in effective elimination of the risk of spoilage due to the growth of autochthonous heterofermentative LAB, as observed by the lack of swollen packs and/or slime production on the product. Therefore, according to the obtained data and despite the fact that cooked cubed ham did not show pH ≤ 4.4 or Aw ≤ 0.92, or pH ≤ 5.0 and Aw ≤ 0.94, as cited in the EC Regulation 2073/2005 [53], it can be scientifically stated that cubes of cooked ham with the addition of bioprotective starters cultures do not constitute a favourable substrate for *L. monocytogenes* growth. Consequently, these products can easily fall into category 1.3 (ready-to-eat foods that are not favourable to *L. monocytogenes* growth, other than those for infants and for special medical purposes), in which a maximum concentration of *L. monocytogenes* of 100 CFU g^−1^ is allowed. Finally, the use of the starters is also suggested because they do not change the sensorial quality of cooked cubed ham.

## Figures and Tables

**Table 1:** Physico-chemical characteristics of the cubed cooked ham.

Parameters	%
Moisture	73.4 ± 0.4
Proteins	18.5± 1.0
Lipids	3.7 ± 0.3
Ash	3.2 ± 0.2
Sugar	1.2 ±1.1
a_w_	0.992 ± 0.002
pH	6.35 ± 0.11

**Table 2:** Presence of *L. monocytogenes* in cooked ham cubes packaged in Modified Atmosphere Packaging (MAP) and stored at 4 °C. Samples were randomly collected at retail.

Brands	1		2		3	
Days	0	60	0	60	0	60
Lot 1	0/5	0/5	0/5	1/5	0/5	0/5
Lot 2	0/5	0/5	0/5	0/5	0/5	1/5
Lot 3	0/5	0/5	0/5	0/5	0/5	1/5
Lot 4	0/5	1/5	1/5	0/5	1/5	0/5
Lot 5	0/5	0/5	0/5	1/5	0/5	0/5
Lot 6	0/5	1/5 *	0/5	0/5	0/5	0/5
Total	0/30	2/30	1/30	2/30	1/30	2/30

Legend: Presence < 100 CFU g^−1^; * presence: 2.1 log CFU g^−1^.

**Table 3:** a_w_ values in cubed cooked ham stored at 4 °C, at 4 °C for 20 days and then at 8 °C for 40 days (end of shelf-life).

Days	a_w_ (4 °C)	a_w_ (4 → 8 °C) *
0	0.986 ± 0.001 aw	0.986 ± 0.001 aw
10	0.992 ± 0.002 av	0.992 ± 0.002 av
20	0.994 ± 0.003 av	0.994 ± 0.003 av
30	0.990 ± 0.001 av	0.986 ± 0.001 bw
40	0.990 ± 0.003 av	0.997 ± 0.001 bv
50	0.993 ± 0.004 av	0.983 ± 0.003 bw
60	0.993 ± 0.001 av	0.992 ± 0.001 av

Legend: * 20 days at 4 °C and 40 more days at 8 °C. Data were represented as the mean ± standard deviation; Different letters (a and b) indicate significant differences between the two temperatures (*p* < 0.05); the other set of different letters (v trough z) indicate differences among a_w_ within each temperature (*p* < 0.05).

**Table 4:** Trend of pH in cubed cooked ham with or without starter addition.

	Control NI		BOX-74		BOX-57	
Days	pH 4 °C	* pH 4 → 8 °C	pH 4 °C	* pH 4 → 8 °C	pH 4 °C	* pH 4 → 8 °C
0	6.35 ± 0.11 av	6.35 ± 0.11 av	6.35 ± 0.11 av	6.35 ± 0.11 av	6.35 ± 0.11 av	6.35 ± 0.11 av
10	6.35 ± 0.20 av	6.35 ± 0.20 av	6.21 ± 0.31 av	6.21 ± 0.31 av	6.18 ± 0.20 av	6.25 ± 0.10 av
20	6.06 ± 0.11 aw	6.06 ± 0.11 aw	6.01 ± 0.21 av	6.01 ± 0.21 av	6.00 ± 0.11 av	5.90 ± 0.25 aw
30	5.82 ± 0.06 ax	5.94 ± 0.08 bw	5.82 ± 0.06 aw	5.94 ± 0.08 bv	5.80 ± 0.06 aw	5.75 ± 0.03 cx
40	6.12 ± 0.06 aw	6.06 ± 0.06 aw	6.02 ± 0.06 av	6.06 ± 0.03 av	5.80 ± 0.10 bw	5.70 ± 0.01 cx
50	6.33 ± 0.05 av	6.27 ± 0.01 bv	5.59 ± 0.25 cw	5.49 ± 0.07 cw	5.48 ± 0.20 cx	5.65 ± 0.22 dx
60	6.18 ± 0.05 ax	6.17 ± 0.02 ax	5.69 ± 0.10 bw	5.14 ± 004 cx	5.65 ± 0.30 bx	5.33 ± 0.20 dy

Legend: Control NI, cubed cooked ham without inoculated starter; BOX-74, trial inoculated with BOX-74 starter only; BOX-57, trial inoculated with BOX-57 starter only; * 20 days of storage at 4 °C followed by additional 40 days at 8 °C. Data were reported as the mean ± standard deviation; Mean with different letters (a versus d) within the lines were significantly different (*p* < 0.05); the other set of different letters (v trough z) indicate differences among pH within each temperature and each treatments (NI, BOX-74 and BOX-57) (*p* < 0.05).

**Table 5:** Colour changes in the different trials stored at different temperatures.

	L* (4 °C)			L* (4 → 8 °C)		
**Days**	NI	BOX-74	BOX-57 *	NI	BOX-74	BOX-57
0	62.86 ± 2.90 av	65.71 ± 1.43 av	65.97 ± 0.72 av	62.86 ± 2.90 av	63.40 ± 5.39 av	65.31 ± 0.79 av
60	65.02 ± 2.81 av	64.66 ± 2.77 av	66.83 ± 0.58 av	65.72 ± 1.03 av	65.74 ± 2.03 av	67.22 ± 1.03 av
	a* (4 °C)			a* (4 → 8 °C)		
	NI	BOX-74	BOX-57 *	NI	BOX-74	BOX-57
0	14.20 ± 0.23 av	14.84 ± 0.32 av	14.24 ± 0.40 av	14.20 ± 0.23 av	13.98 ±1.15 av	14.65 ± 0.18 av
60	14.23 ± 0.65 av	14.29 ± 0.31 av	14.65 ± 0.11 av	14.26 ± 0.35 av	14.57 ±0.22 av	13.23 ± 0.61 av
	b* (4 °C)			b* (4 → 8 °C)		
	NI	BOX-57	BOX-57	NI	BOX-57	BOX-57
0	3.60 ± 0.50 av	3.66 ± 0.13 av	3.51 ± 0.20 av	3.60 ± 0.50 av	3.07 ± 0.74 av	3.62 ± 0.10 av
60	3.59 ± 0.28 av	3.36 ± 0.12 av	3.51 ± 0.26 av	4.36 ± 0.56 av	3.76 ± 0.23 av	3.48 ± 0.45 av

Legend: Control NI, cubed cooked ham without inoculated starter; BOX-74, trial inoculated with BOX-74 starter only; BOX-57, trial inoculated with BOX-57 starter only; * 20 days of storage at 4 °C, followed by additional 40 days at 8 °C. Data were reported as the mean ± standard deviation; mean with different letters (a versus d) within the lines were significantly different (*p* < 0.05); the other set of different letters (v trough w) indicate differences among days within each temperature and each treatments (NI, BOX-74 and BOX-57) (*p* < 0.05).

**Table 6:** Fate of *L. monocytogenes* coinoculated with BOX-74 and BOX-57 starter cultures in cubed cooked ham stored in MAP at 4 °C, at 4 °C for 20 and then for 40 days at 8 °C.

Temperature 4 °C					
4 → 8 °C					
Days	* LM	BOX-74 + LM	BOX-57 + LM	BOX-74 + LM	BOX-57 + LM
0	2.1 ± 0.3 av	2.1 ± 0.1 av	2.1 ± 0.1 av	2.1 ± 0.1 av	2.1 ± 0.1 av
10	2.8 ± 0.1 bw	2.2 ± 0.2 av	2.3 ± 0.1 av	2.2 ± 0.2 av	2.3 ± 0.1 av
20	4.9 ± 0.7 ax	2.2 ± 0.4 bv	1.6 ± 0.2 cw	2.2 ± 0.4 bv	1.6 ± 0.2 cw
30	8.2 ± 1.3 ay	1.7 ± 0.4 bv	1.6 ± 0.1 bw	1.9 ± 0.3 bv	1.4 ± 0.1 bcx
40	7.9 ± 0.5 ay	1.5 ± 0.5 bv	1.3 ± 0.2 bw	1.6 ± 0.5 bv	1.1 ± 0.5 by
50	8.5 ± 0.2 ay	1.5 ± 0.2 bv	1.3 ± 0.1 bw	1.5 ± 0.1 bv	1.3 ± 0.2 bx
60	8.4 ± 0.3 ay	1.8 ± 0.2 bv	1.2 ± 0.4 cw	1.9 ± 0.3 bv	1.4 ± 0.1 cx

Legend: Data were reported as the mean ± standard deviation; mean with different letters (a versus e) within the lines were significantly different (*p* < 0.05); the other set of different letters v trough z) indicate differences among days within each treatments (* *Listeria monocytogenes* (LM), and BOX-74 or BOX-57 starter inoculated with *L. monocytogenes*) (*p* < 0.05).

**Table 7:** Sensorial evaluation by untrained assessors.

Parameter	Control	Starter 1	Starter 2
Fermented	0/12	0/12	0/12
Rancidity	0/12	0/12	0/12
Sweet	10/12	9/12	9/12
Acid	0/12	1/12	1/12
Meaty	12/12	12/12	12/12
Fresh	6/12	6/12	7/12
Bitter	0/12	0/12	0/12
Ammoniacal	0/12	0/12	0/12
Slime	0/12	0/12	0/12
Final value *	**2**	**2**	**2**

* Scores: 1 (excellent). 2 (good). 3 (sufficient). 4 (scarse).
